# Virtual Multidisciplinary Gastrointestinal Care for Adults With Gastrointestinal Needs: Retrospective Cohort Study

**DOI:** 10.2196/89061

**Published:** 2026-04-23

**Authors:** Grace Wang, William D Chey, Sanskriti Varma, Sameer Berry

**Affiliations:** 1 Oshi Health, Inc. New York, NY United States; 2 Department of Internal Medicine Division of Gastroenterology University of Michigan Ann Arbor, MI United States; 3 Massachusetts General Hospital Boston, MA United States; 4 New York University New York, NY United States

**Keywords:** gastroenterology, patient care team, telemedicine, advanced practice provider, disorders of the gut-brain interaction

## Abstract

**Background:**

Gastrointestinal (GI) disorders are highly prevalent, costly, and complex. Multidisciplinary gastrointestinal care (MGC), integrating medicine, nutrition, and behavioral health, is a best practice for managing GI needs, but access is limited by the availability of gastroenterologists and MGC clinics. Virtual MGC may bridge the gap, but it is unclear the extent to which patients engage in virtual MGC and the outcomes of virtual services delivered at scale.

**Objective:**

This study describes patient characteristics, engagement, and outcomes of a large-scale virtual MGC program and evaluates whether dietitian and behavioral health support mediates the association between medical engagement and symptom improvement.

**Methods:**

We conducted a retrospective cohort study of 11,345 adult patients receiving virtual MGC with gastroenterologists, GI-specialized advanced practice providers (APPs), registered dietitians, licensed psychologists, and care coordinators between April 2021 and August 2025. Patients completed virtual onboarding with a GI APP and medical, dietary, and behavioral interventions delivered through synchronous telehealth and asynchronous chat. Data were from web-based intake forms and electronic health records. Patient-reported outcomes included symptom control and symptom improvement (Yes/No). Descriptive analyses, logistic regression, and path analysis were conducted across 20 imputed datasets.

**Results:**

Virtual patients receiving MGC were 43.3 (SD 12.78) years on average, and 66.4% (7532/11,345) were female. Patients primarily presented with Disorders of Gut-Brain Interaction (4460/11,345, 39.3%) and Gastroesophageal Reflux Disease (2775/11,345, 24.5%). The median wait time for an initial appointment was 6 days (IQR 3-9). Patients had a median of 2 GI visits (IQR 1-3), 2 dietitian visits (IQR 1-3), and 1 behavioral health visit (IQR 0-2). As the number of visits increases, the odds of achieving positive outcomes increase significantly after controlling for age, sex, symptom severity at baseline, symptom frequency at baseline, and days in care. This translates to 92.39% with symptom improvement, 94.67% with symptom control, and 98.13% with no noticeable or mild symptoms among those with 4 or more appointments. Path analysis confirmed that GI APP engagement was significantly associated with increased dietitian and behavioral health usage, which was associated with symptom improvement. The direct pathway from GI APP engagement to symptom improvement was also significant.

**Conclusions:**

This innovative study demonstrates that a virtual-first MGC model is not only feasible at a national scale but is effective in achieving symptom control and improvement across a clinically diverse GI population. We provide evidence about successfully delivering high-quality care outside traditional clinical settings. This work distinguishes itself by analyzing the mechanisms of integrated care, specifically how medical engagement facilitates the use of specialized nutritional and behavioral interventions that are often inaccessible in community care. In the real world, this model offers a scalable solution to geographic specialist shortages, ensuring that best-practice care is available to patients regardless of their location or the local supply of specialists.

## Introduction

### Problem and Review of Relevant Scholarship

Gastrointestinal (GI) disorders are highly prevalent, decrease patients’ quality of life, and incur considerable cost to the US health system. About 6 out of 10 adults report GI symptoms every week [1]. Many patients experience shame, high rates of absenteeism, and disruptions to their social life [2,3]. In 2021, GI-related health care expenditures totaled US $111.8 billion from medical services, including 14.5 million emergency department visits and 2.9 million hospital admissions [4]. GI-related health care expenditure exceeds that of mental health, heart disease, and breast and prostate cancers [5-7].

Because GI disorders can be complex with considerable symptom and diagnosis overlap, multidisciplinary approaches are increasingly recognized as a new best practice. Multidisciplinary gastrointestinal care (MGC) integrates therapies from medicine, nutritional science, and behavioral health to offer a holistic approach to GI needs [8]. In a randomized controlled trial, a multidisciplinary care model showed superior efficacy, with 84% of participants reporting global symptom relief compared with only 57% of those in the conventional care arm [9]. These advantages endured; at 1 year post treatment, patients in the multidisciplinary group were nearly twice as likely as conventional care patients to report their symptoms as improved [10]. Furthermore, evidence from a matched cohort study confirms that the multidisciplinary approach yields significantly better outcomes than standard care [11]. Reflecting this growing evidence base, current clinical guidelines from the American College of Gastroenterology now incorporate gut-directed psychotherapies as a primary recommendation for managing global irritable bowel syndrome symptoms [12].

Despite this clinical consensus, in-person MGC is not yet widespread. There are profound geographic disparities in access to gastroenterologists across the United States. Recent research notes that 39% of 1167 metropolitan counties and 87% of 1975 nonmetropolitan counties had no gastroenterologists [13]. Even in many major metropolitan areas, the average wait time for an in-person gastroenterology appointment reached 40 days in 2025 [14]. While in-person MGC clinics exist, they are typically offered by academic medical centers, leaving the vast majority of the population without access to integrated dietary and behavioral support. Furthermore, referral processes to dietitians and behavioral health providers and reimbursement approaches for MGC can be greatly improved [15,16].

Virtual MGC models have been proposed to bridge this gap, with evidence suggesting that telehealth is effective for managing GI needs and highly acceptable to patients [17,18]. In response, we designed a virtual MGC program with access to board-certified gastroenterologists, GI-specialized advanced practice providers (APPs), registered dietitians, licensed psychologists, and care coordinators. The program’s objective is to provide access to high-quality MGC that patients nationwide need, wherever they are. However, it is unclear the extent to which patients engage in virtual MGC and the magnitude of outcomes that can be achieved with virtual delivery of services at scale.

### Aims and Objectives

To address these gaps, our aim was to describe (1) the patients who received virtual MGC, (2) patient engagement with virtual MGC, (3) symptom improvement experienced by patients and satisfaction with services, and (4) the relationship between engagement and symptom improvement among virtual patients receiving MGC. The second aim was to examine whether the association between GI engagement and symptom improvement is mediated by the activation of specialized dietitian and behavioral health services. To achieve these aims, our objective was to conduct an observational study with more than 11,000 patients with GI conditions who received virtual MGC.

## Methods

This manuscript follows Journal Article Reporting Standards reporting guidelines [19].

### Inclusion and Exclusion

We included patients who met all of the following: ≥18 years of age at enrollment; reside in the United States; have access to internet or mobile technology; English speaking; provided intake data; had their first onboarding appointment with an APP between April 2021 through August 2025; and were referred to and proceeded to virtual MGC (ie, care with the broader GI, behavioral health, and dietician team).

### Participant Characteristics

This study includes 11,345 virtual patients receiving MGC. Of these, 7532 (66.39%) were female. The average age was 43.27 (SD 12.78) years, and patients lived in all 50 US states and the District of Columbia.

### Sampling Procedures and Setting

The study included all patients meeting the inclusion criteria. Virtual MGC includes board-certified gastroenterologists, GI-specialized APPs, registered dietitians, licensed psychologists, and care coordinators. Access was through 3 primary channels: health plan networks, corporate benefits, and external provider referrals. Eligible patients were informed about virtual MGC services via email or mailings directing them to a web-based registration system. After completing an intake questionnaire and providing demographic details, patients self-schedule a virtual onboarding visit with a GI APP.

The care team requests and reviews past medical records before the first visit. The APP identifies a preliminary diagnosis and provides a written care plan, which may include diagnostic testing, procedures, over-the-counter or prescription medications, dietary interventions, and brain-gut behavioral therapies. GI APPs follow evidence-based protocols to diagnose, treat, and prescribe independently, depending on the patient’s state of residence; gastroenterologists on the care team are available for consultation and verification. The care plan is available to patients and other care team members in the patient’s electronic health record (EHR). Patients also receive tracking tools and educational resources.

A subset of patients is offered and enter multidisciplinary care with a medical, dietary, and behavioral health care team who monitors and may revise the initial treatment plan according to patient needs. Multimedia Appendix 1 lists example interventions offered by the care team. Patients have as many virtual visits with care team members as needed until achieving symptom control.

Synchronous telehealth visits are via the clinic’s digital platform, which also offers asynchronous chat messaging with care teams for support in between visits or calls. When in-person services (eg, endoscopy and imaging) are warranted, the care team refers patients to in-network partner facilities, and care coordinators support patients with referrals.

### Sample Size, Power, and Precision

The sample size was determined by the total number of eligible patients who received virtual MGC between April 2021 and August 2025 and met the inclusion criteria. As this was a retrospective cohort study using real-world data, no a priori power analysis was conducted. The final analytical sample included 11,345 patients. For primary binary outcomes, the large sample size yielded narrow 95% CIs around effect estimates (see Results), indicating high statistical precision. Given the observed outcome rates, the available sample provided adequate power (≥0.80 at α=.05) to detect small effect sizes in multivariable logistic regression models.

### Measures

This analysis included the following variables.

#### Baseline Patient Characteristics

Demographic variables included age, sex, state of residence, GI conditions and symptoms, and comorbidities at baseline. We also examined 3 symptom items at baseline: symptom severity (1 not noticeable [no disruption to daily life], 2 mild [minimal disruption to daily life], 3 moderate [disruptive to daily life], 4 severe [very disruptive to daily life]), number of symptoms, and symptom frequency (1 day per month or less, 2-3 times per month, 1-2 days per week, 3-4 days per week, 5-6 days per week, 7 days per week).

#### Engagement

Metrics include: time from account creation to first onboarding appointment with an APP and number of visits per patient overall and by appointment type (ie, medical, dietary, or behavioral). Duration was measured using visit minutes and call minutes per patient by appointment type. To exclude voicemails, we included calls lasting 5 minutes or more. For chats, we report on the number of messages initiated by patients.

#### Patient-Reported Outcomes

Symptom Control was measured by, “Do you feel [the virtual MGC] has helped you gain control over your GI condition or symptoms during the course of this program?” (Yes/No). When patients provided multiple responses over time to these items, this analysis included the best outcome achieved.

To create Symptom Improvement (0=no improvement, 1=improvement), we recategorized patient responses to the question, “Compared to before I was seen by [virtual MGC], my GI condition is now: worse, slightly worse, same, slightly better, better.” Those who responded worse, slightly worse, or the same were categorized as no improvement. Those who responded slightly better or better were categorized as improved. For those with missing responses to this question, we examined baseline symptom severity compared with symptom severity score after receiving virtual MGC. Symptom severity responses were 1 not noticeable (no disruption to daily life), 2 mild (minimal disruption to daily life), 3 moderate (disruptive to daily life), 4 severe (very disruptive to daily life). Those whose scores decreased by 1 or more points were categorized as 1 on Symptom Improvement, else 0.

Symptom Severity was transformed into a binary variable with categories not noticeable or mild (0), or moderate or severe (1).

#### Satisfaction

Participants answered, “How satisfied were you with your prior GI care?” at baseline and, “How satisfied were you with your recent [virtual MGC] experience?” after each visit. Responses were: 1 not satisfied at all, 2 somewhat dissatisfied, 3 neither satisfied nor dissatisfied, 4 somewhat satisfied, 5 very satisfied, or I have not previously worked with a GI provider (baseline only). The average score was used for those who provided multiple responses after multiple visits.

### Data Collection

Data were collected in the following ways.

- Intake forms: Patients completed intake forms via the web before their first onboarding appointments with GI APPs. Patient characteristics and symptom severity were self-reported through intake forms.
- EHR and Health Cloud data systems: *ICD-10* (*International Statistical Classification of Diseases, Tenth Revision*) and SNOMED codes were gathered from EHRs and used to describe patients’ GI-specific conditions, symptoms, and comorbidities. Patient-reported outcomes (PROs) were recorded in Health Cloud (Salesforce) by providers during visits.
- Scheduling systems: Engagement data were gathered from the scheduling system.
- Patient surveys: Satisfaction was collected as an electronic survey after each visit.

### Quality of Measurements

To ensure data collection quality, intake forms included data-type constraints (eg, restricted numeric entry for age) to prevent erroneous entries. At the clinical level, all providers are trained to collect outcomes uniformly.

### Instrumentation and Psychometrics

Patient outcomes were assessed using modified Patient Global Impression scales for symptom severity, improvement, and control. In this study, the scales focused on virtual MGC and used a 4-point scale for symptom severity, a 5-point scale for improvement, and a binary scale for control. These instruments are widely used single-item global ratings designed to capture patients’ subjective evaluation of symptom severity and perceived improvement over time. They have demonstrated face validity, construct validity, responsiveness to clinical change, and interpretability across chronic conditions [20-23]. They have been used as anchors for psychometric testing of other scales in GI research [24-26].

### Masking

Due to the retrospective, single-arm observational design of this study, masking of participants and providers was not applicable. All patients were aware they were receiving virtual MGC as part of their clinical treatment plan, and providers were aware of the collection of clinical data and PROs as part of their routine care delivery.

### Conditions and Design

We conducted a nonexperimental, retrospective cohort study of patients who first received virtual MGC between April 2021 and August 2025.

We used Andersen’s model [27] of health care usage to guide variable selection for the analysis of the relationship between engagement and symptom improvement (Multimedia Appendix 2).

### Data Diagnostics

Before analysis, all 41 variables were screened for outliers using distributional inspection; all observed values were determined to be plausible and were retained. The overall proportion of missing data across all 41 variables was 3.0% (14,077 of 465,145 data points). Little’s test indicated that data were not missing completely at random (*χ*^2^_688_=5013.97, *P*<.001) [28]. Based on missingness patterns supporting the assumption that data were missing at random, we performed multiple imputation by chained equations. Twenty imputed datasets were generated, and estimates were pooled using Rubin’s rules. All generalized structural equation modeling (GSEM) and logistic regression models achieved convergence across the 20 imputed datasets. The number of missing and imputed values for all model variables is provided in Multimedia Appendix 3.

### Analytic Strategy

We calculated descriptive statistics across 20 pooled imputed datasets for all patient characteristics, engagement, PRO, and satisfaction variables.

To examine associations between engagement and outcomes (symptom improvement and symptom control), we conducted logistic regression modeling across 20 imputed datasets. Adjusted models controlled for age, sex, symptom severity at baseline, symptom frequency at baseline, and days in care. Model discrimination was assessed using the area under the receiver operating characteristic curve in a representative imputed dataset. Following estimation, we used the margins command on the pooled results to calculate the predicted probabilities of symptom improvement and symptom control. These average marginal effects are reported below.

Path analysis was conducted using GSEM within a multiple-imputation framework (20 imputations) to evaluate the mediating role of multidisciplinary specialist engagement (Registered Dietitian and Behavioral Health) on the relationship between GI APP visits and symptom improvement. Models were adjusted for age, sex, symptom severity at baseline, symptom frequency at baseline, presence of disorders of the gut-brain interaction (DGBI), number of days in care, client, and state. Inference was based on multiple-imputation–combined estimates using large-sample (asymptotic) standard errors, such that test statistics follow the standard normal (z) distribution. Statistical significance was assessed using 2-sided tests with α of .05. Indirect effects were estimated using the product-of-coefficients method on the log-odds scale. Because odds ratios (ORs) are noncollapsible, total effects were estimated separately, and the proportion mediated was not calculated.

All analyses were conducted in Stata SE (version 19.5; StataCorp LLC).

### Ethical Considerations

Pearl Institutional Review Board reviewed the study protocol and determined the study to be Exempt according to 45 CFR 46.104(d)(4) Secondary Research Uses of Data or Specimens. This study involves the analysis of data generated during routine clinical care. The institutional review board granted a waiver of informed consent, as the research posed minimal risk to participants and could not practicably be carried out otherwise. To protect participant privacy and confidentiality, all data used in this analysis were deidentified in accordance with the HIPAA (Health Insurance Portability and Accountability Act) Privacy Rule. Direct identifiers were removed, and unique study identifiers were used in place of personal information. Data were stored on secure, access-restricted servers, and only authorized study personnel had access to the analytic dataset. Participants did not receive financial or other compensation for inclusion in this secondary data analysis. This manuscript does not include images, figures, or supplementary materials that could identify individual participants.

## Results

### Patient Flow

**Figure 1:**
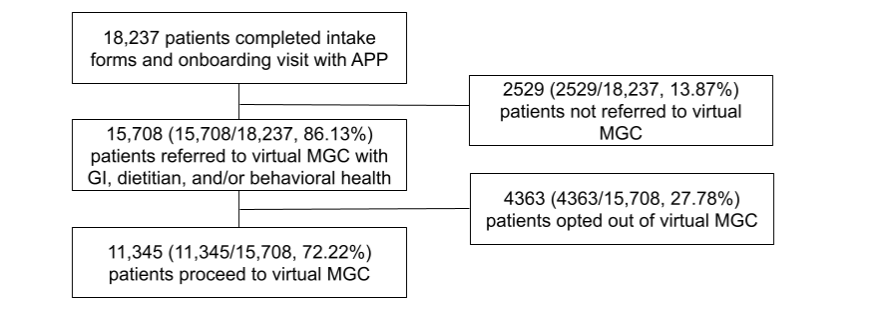
Patient flow from onboarding to virtual MGC for patients with GI needs who received care between April 2021 and August 2025. APP: advanced practice provider; GI: gastrointestinal; MGC: multidisciplinary gastrointestinal care.

Between April 2021 and August 2025, 18,235 patients had a first virtual appointment with a GI APP and provided intake data. Of these, GI APP used their clinical judgment to refer 15,708 to virtual MGC with a registered dietitian or behavioral health provider. Of the referred, 11,345 received virtual MGC (Figure 1).

### Patient Description

Table 1 shows virtual patients receiving MGC characteristics at baseline. The majority (7924/11,345, 69.85%) of patients reported that GI symptom severity was moderate (disruptive to daily life) or severe (very disruptive to daily life). More than half (6275/11,345, 55.30%) experienced symptoms 5 or more days per week.

The most common diagnoses at first appointments for 11,345 patients were DGBI (4460/11,345, 39.31%), gastroesophageal reflux disease (2775/11,345, 24.46%), and inflammatory bowel disease (441/11,345, 3.89%). The most common symptoms reported by 11,345 patients were flatulence (3751/11,345, 33.06%), bloating (3587/11,345, 31.62%), and heartburn or reflux (2887/11,345, 25.45%) (Table 2).

Among 8989 patients with known comorbidities, the average number was 2.27 (SD 1.37) comorbid conditions, with behavioral health (3963/8989, 44.09%), metabolic (3823/8989, 42.53%), and cardiovascular (3595/8989, 39.99%) conditions being the most prevalent.

**Table 1:** Characteristics at baseline for virtual multidisciplinary gastrointestinal care patients who received care between April 2021 and August 2025 (n=11,345).

Characteristics			Value, n (%)
**Sex**			
	Female	7532 (66.39)	
	Male	3813 (33.61)	
**Age at enrollment (years)**			
	18 to 29	1572 (13.86)	
	30 to 39	3281 (28.92)	
	40 to 49	2840 (25.03)	
	50 to 59	2279 (20.09)	
	60 and older	1373 (12.10)	
**Symptom severity at baseline**			
	Not noticeable (no disruption to daily life)	230 (2.02)	
	Mild (minimal disruption to daily life)	3191 (28.13)	
	Moderate (disruptive to daily life)	6212 (54.76)	
	Severe (very disruptive to daily life)	1712 (15.09)	
**Symptom frequency at baseline**			
	1 day per month or less	275 (2.42)	
	2-3 times per month	859 (7.57)	
	1-2 days per week	1423 (12.55)	
	3-4 days per week	2513 (22.15)	
	5-6 days per week	2435 (21.46)	
	7 days per week	3840 (33.84)	

**Table 2:** Ten most prevalent diagnoses and symptoms at baseline from ICD-10 (International Statistical Classification of Diseases, Tenth Revision) codes in the electronic health record among virtual multidisciplinary gastrointestinal care patients who received care between April 2021 and August 2025 (n=11,345)

Characteristic		Value, n (%)
**Diagnoses**		
	DGBI^a^ (including irritable bowel syndrome and other functional disorders)	4460 (39.31)
	GERD^b^	2775 (24.46)
	IBD^c^	441 (3.89)
	Anal or rectal fissures, fistula, or abscesses	283 (2.49)
	Gastroenteritis	257 (2.27)
	Hemorrhoids	229 (2.02)
	Intestinal malabsorption	222 (1.96)
	Diseases of the liver	191 (1.68)
	Small intestinal bacterial overgrowth	180 (1.59)
	Diverticular disease	166 (1.46)
**Symptoms**		
	Flatulence	3751 (33.06)
	Bloating	3587 (31.62)
	Heartburn or reflux hypersensitivity	2887 (25.45)
	Constipation	2254 (19.87)
	Abdominal or pelvic pain	2200 (19.39)
	Diarrhea	1610 (14.19)
	Nausea and vomiting	921 (8.12)
	Change in bowel habits	651 (5.74)
	Chest pain	568 (5.01)
	Fecal abnormalities	499 (4.40)

^a^DGBI: disorders of the gut-brain interaction.

^b^GERD: gastroesophageal reflux disease.

^c^IBD: inflammatory bowel disease.

### Patient Engagement With Virtual MGC

The median number of days for patients to schedule their first GI APP appointment was 6 (IQR 3-9) days. The median number of days from the first GI APP appointment to an appointment with either a registered dietician or behavioral health provider was 15 (IQR 7-28) days.

The median number of visits with GI providers was 2 (IQR 1-3), while registered dietitians and behavioral health providers saw a median of 2 (IQR 1-3) and 1 (IQR 0-2) visits, respectively. On average, GI appointments were 59.31 (SD 45.14) minutes, and calls were 25.16 (SD 25.05) minutes. Behavioral health appointments were 38.85 (SD 52.05) minutes, and calls were 31.07 (SD 22.11) minutes. Dietitian appointments were 64.42 (SD 122.35) minutes, and calls were 29.51 (SD 18.99) minutes. On average, patients initiated 8.36 (SD 17.83) chat messages with their care team.

### Clinical Outcomes

**Figure 2:**
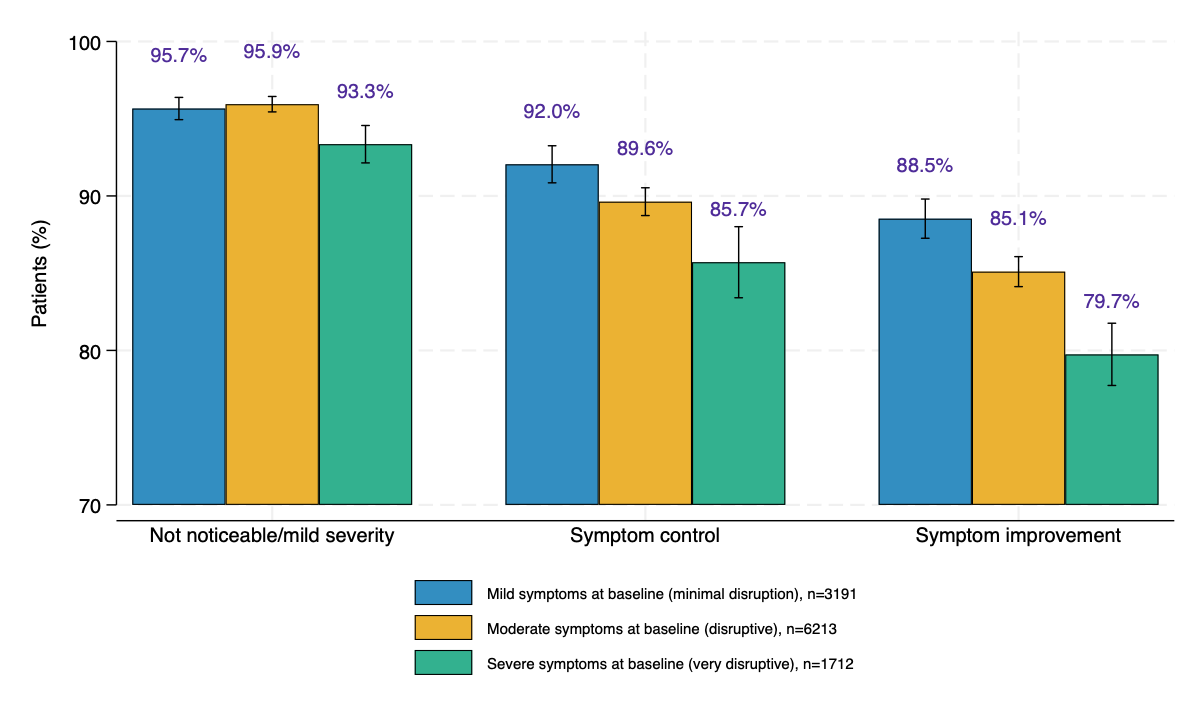
Percent of virtual multidisciplinary gastrointestinal care patients who achieved symptom improvement, symptom control, and no noticeable or mild symptoms after virtual multidisciplinary gastrointestinal care, by baseline symptom severity.

Of the 11,345 patients, 9683 (85.4%) patients reported symptom improvement over time, and 10,189 (89.8%) patients reported symptom control. Median time to symptom control was 29 days. More than 95% (10,803/11,345) reported that symptom severity posed minimal to no disruption to daily life after virtual MGC. Figure 2 reports symptom improvement, control, and severity stratified by baseline symptom severity.

### Satisfaction With Services

We examined care satisfaction scores for 7596 patients who previously saw an in-person provider for their GI needs. Mean satisfaction scores increased significantly from 2.74 (SD 1.31) at baseline to 4.82 (SD 0.61) after virtual MGC.

### Association Between Engagement and Outcomes

**Figure 3:**
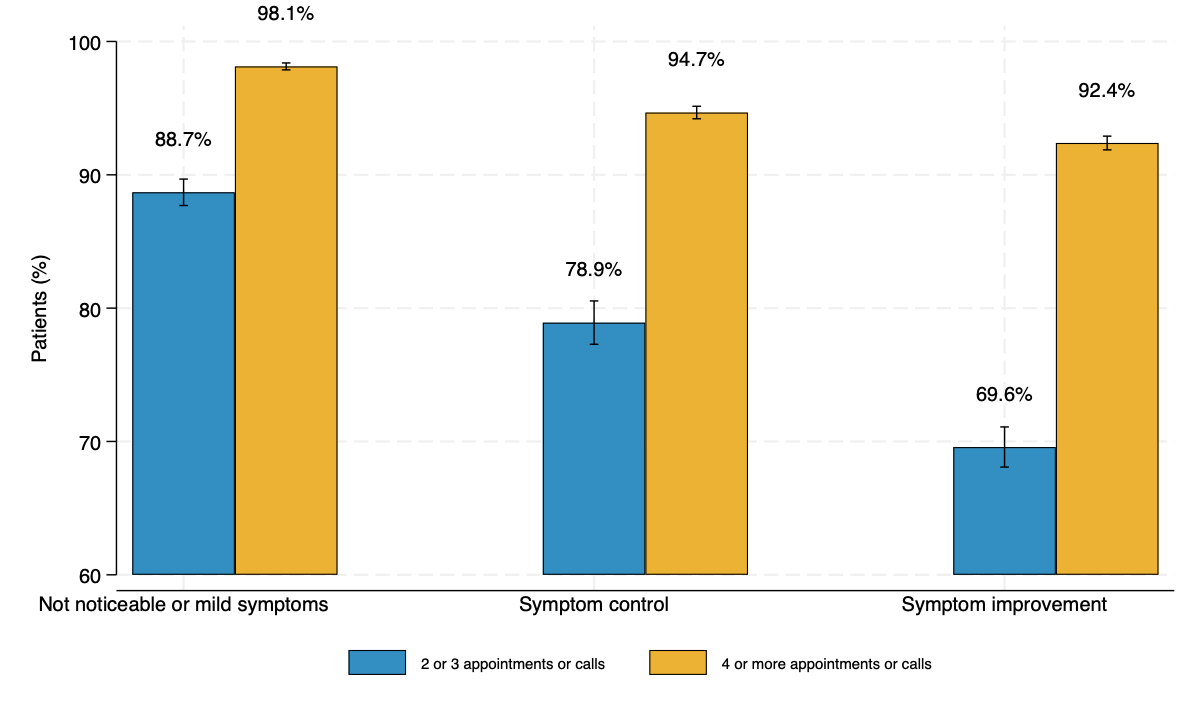
Predicted probability of virtual multidisciplinary gastrointestinal care patients achieving positive outcomes (symptom improvement, symptom control, not noticeable or mild symptoms), by number of appointments or calls with the care team. Estimates control for age, sex, symptom severity at baseline, symptom frequency at baseline, and days in care (n=11,345).

As the number of visits increases, the odds of achieving positive outcomes increases after controlling for age, sex, symptom severity at baseline, symptom frequency at baseline, and days in care (symptom improvement: OR 1.51, 95% CI 1.44-1.58; symptom control: OR 1.46, 95% CI 1.38-1.55; not noticeable or mild symptoms: OR 1.91, 95% CI 1.77-2.06). For example, Figure 3 shows the predicted probability of symptom improvement is 69.58% for patients with 2 or 3 appointments versus 92.39% with 4 or more appointments.

### GI APP Engagement and Symptom Improvement, Mediated by Dietitian and Behavioral Health Support

**Figure 4:**
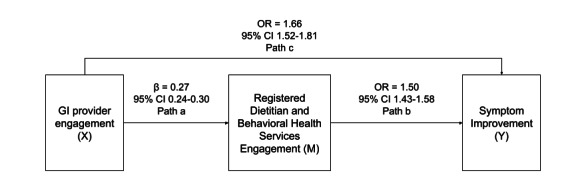
Path analysis of the multidisciplinary care model. Standardized coefficients (beta) and odds ratios (OR) are presented for the direct and mediated pathways. Models adjust for age, sex, baseline symptom severity, baseline symptom frequency, symptom number, DGBI diagnosis, employer, and state fixed effects. Estimates are pooled from 20 imputed datasets (n=11,345).

Path analysis using GSEM (Figure 4) showed that GI APP appointments were significantly associated with an increase in multidisciplinary care team appointments with registered dietitians and behavioral health providers (path a; *β*=0.27, 95% CI 0.24-0.30; *z*=18.04, *P*<.001). In turn, these specialized multidisciplinary services were significantly and positively associated with symptom improvement (path b; OR 1.50, 95% CI 1.43-1.58; *z*=16.22, *P*<.001). The direct pathway from GI APP engagement to symptom improvement (path c) remained statistically significant after adjustment for the multidisciplinary mediator (OR 1.66, 95% CI 1.51-1.80; *z*=22.54, *P*<.001). In the reduced model excluding the mediator, the total effect of GI APP appointments on symptom improvement was also statistically significant (OR 1.50, 95% CI 1.37-1.62; *z*=23.09, *P*<.001). All pathways were adjusted for age, sex, baseline symptom severity and frequency, symptom count, DGBI diagnosis, employer, and state fixed effects.

## Discussion

### Principal Results

This observational study reports the performance of virtual MGC at a national scale for more than 11,000 patients. Our first aim was to describe (1) the patients who received virtual MGC, (2) patient engagement with virtual MGC, (3) symptom improvement experienced by patients and satisfaction with services, and (4) the relationship between engagement and symptom improvement among virtual patients receiving MGC. We show that a virtual MGC model is able to serve patients from across the United States, with a range of GI needs, and experiencing mild to severe symptoms. Patients all received care by GI APPs, and most had multiple and extensive interactions with behavioral and nutritional health care team members. Patient symptom severity improved significantly over time, and the vast majority of patients reported that they were in control of their symptoms. Patients reported high satisfaction with care, and engagement with the multidisciplinary care team was significantly associated with positive outcomes. The second aim was to examine whether the association between GI engagement and symptom improvement is mediated by the activation of specialized dietitian and behavioral health services. Path analysis confirmed that the association between GI APP engagement and symptom improvement is significantly mediated by the activation of specialized nutritional and behavioral services.

### Similarity of Results

The clinical outcomes in this study are consistent with an RCT by Basnayake et al [9], which reported that 85% of patients in an in-person MGC group achieved symptom improvement compared with only 65% of patients in the standard gastroenterologist-only care group. These findings suggest that virtual MGC, when delivered in real-world settings, can achieve similar positive outcomes as those observed in a controlled clinical trial.

### Interpretation

Patient satisfaction with virtual GI services was high, which was consistent with previous studies on virtual GI care [29-31]. We hypothesize that this high satisfaction stems from patients’ preference for the convenience of virtual visits and perceptions that the quality of care is comparable to in-person services. A 2024 national survey of patients who used telehealth supported this notion, with 86% agreeing that “telehealth made it easier for me to get care when and where I needed it” and 78% agreeing that “the care I received through telehealth was as good as a regular in-person visit” [32].

Our study found that engagement with the multidisciplinary team was a meaningful pathway in the relationship between GI APP and symptom improvement. This positive relationship between MGC and outcomes is compatible with clinical trial results and evidence-based practice guidelines that support MGC [10,33-35]. The ongoing support from registered dieticians meets patients’ needs for guidance about nutrients, adequate caloric intake, and appropriate diet interventions tailored to the patient’s lifestyle [36]. Access to the dietitian over time is crucial for determining any needed modifications to the recommended diet and supporting adherence to the diet [37].

Similarly, ongoing support from behavioral health providers helps patients learn and refine approaches for managing the psychosocial factors that can trigger their conditions. Behavioral health can help with coping mechanisms for GI-specific symptoms and facilitate rewiring of neuronal inputs, which leads to improved functional disorders [12,38-40]. GI-focused behavioral therapies exert therapeutic effects through bidirectional modulation of the brain-gut axis, targeting not only central processes such as mood and cognitive appraisal, but also peripheral mechanisms including pain perception, visceral hypersensitivity, and GI motility [12,38-40]. Furthermore, behavioral health providers help many patients with anxiety and depression exacerbated by GI conditions. Together, the team is able to evaluate the benefits and risks of food restrictions on the patient’s GI and psychological needs [41].

### Generalizability

The demographics of the virtual patients receiving MGC base were similar to those reported by Almario et al [1] in their nationally representative study of GI symptoms in the US population. Almario et al [1] found that 52% of adults with GI symptoms were 25 to 44 years old, and 60% were female, which aligns with the age and sex distribution observed in our cohort. Furthermore, the most common GI conditions for virtual patients receiving MGC (ie, DGBI, gastroesophageal reflux disease, and inflammatory bowel disease) correspond to the most prevalent symptoms (eg, bloating, heartburn or reflux, abdominal or pelvic pain, diarrhea, and constipation) reported by Almario et al [1]. This consistency suggests that the patient population seeking virtual MGC is broadly representative of individuals experiencing GI issues in the general US population.

### Implications

The findings of this study have implications for research, systems, and policy. Future research could focus on identifying the minimum effective dose of multidisciplinary touchpoints required to achieve and sustain positive outcomes. Prospective trials comparing virtual MGC directly to traditional community-based care are warranted to further quantify its impact on clinical outcomes and health care usage. Research is also needed to evaluate alternative payment models that best support virtual MGC.

For health systems and policy makers, virtual care delivery may be an important approach for addressing provider shortages and associated delays in care. This is underscored by our finding that virtual patients receiving MGC waited a median of only 6 days for a first visit, in contrast to the average 40-day wait time for an in-person gastroenterologist visit in major metropolitan areas in 2025 [14]. In addition, our results support more emphasis on integrated clinical ecosystems with medical providers directing care and coordinating with dietitians and behavioral providers. This virtual-first, integrated model may serve as a blueprint for value-based care programs designed to improve patient quality of life and reduce downstream costs associated with GI needs. Reimbursing by milestones instead of traditional fee-for-service billing would allow patients to have as many visits as needed with any provider type.

### Strengths and Limitations

A strength of this study is the large sample size of >11,000 patients from across the United States and with a range of GI conditions. This demonstrates the feasibility of delivering virtual MGC at scale, and the diverse patient base supports the generalizability of findings to a broad US patient population. Our study is also the first, to our knowledge, to demonstrate the role of nutritional and behavioral support in mediating the association between GI medical care and symptom improvement within a virtual MGC setting.

Our study has a number of limitations, including the absence of a control or comparison group, as our analysis only includes individuals who received virtual MGC. Therefore, we cannot rule out the possibility that the natural history of the GI conditions, which are often relapsing and remitting, may have contributed to the observed symptom improvements. Future research should compare to a control group not receiving virtual MGC or receiving only in-person care.

Our analysis did not include variables such as patient health behaviors or in-person health care usage, which could influence symptom improvement. A future study using medical claims could incorporate important factors, such as diagnostic testing, procedures, and prescriptions. Another limitation is our use of global impression scales to capture symptom improvement, control, and severity. Global impression scales minimize patient burden and are practical for large-scale observational research [42]. However, these scales do not capture the granular information about symptoms like other validated measures, such as Patient-Reported Outcomes Measurement Information System (PROMIS) GI scales [43,44]. Future studies incorporating both global impression and PROMIS scales could be used to more comprehensively evaluate virtual MGC.

### Conclusions

This innovative study demonstrates that a virtual-first MGC model is not only feasible at a national scale but is effective in achieving symptom control and improvement across a clinically diverse GI population. Furthermore, we demonstrate that high-quality, evidence-based GI care can be successfully delivered outside traditional brick-and-mortar settings. It differs from existing literature by moving beyond feasibility and effectiveness to provide an analysis of how medical engagement actively drives the use of specialized nutritional and behavioral interventions, which are often difficult services for patients to access in community settings. This work brings a new understanding to the field regarding the role of medical providers in integrated teams. Care effectiveness is amplified when medical management serves as a gateway to integrated nutritional and behavioral interventions. In the real world, health systems that develop virtual capabilities can greatly improve access to best-practice care for patients, no matter the supply of specialists in the region. Ultimately, virtual MGC is a responsive, patient-centered model that addresses the physical, nutritional, and psychological complexities of GI health.

## Appendix

Multimedia Appendix 1 Example dietary and behavioral health interventions offered through virtual MGC.

Multimedia Appendix 2 Andersen’s model of health care utilization. This model shows the relationship between patient characteristics (i.e., predisposing, enabling, and need factors) and health behaviors, including service utilization. In turn, the health behaviors influence outcomes, such as symptom improvement and symptom control.

Multimedia Appendix 3 Number and percent of complete and imputed values, by characteristic, for the 11,345 virtual MGC patients who received care between April 2021 and August 2025.

## Abbreviations

APP: advanced practice provider
DGBI: disorders of the gut-brain interaction
EHR: electronic health record
GI: gastrointestinal
GSEM: generalized structural equation modeling
HIPAA: Health Insurance Portability and Accountability Act
ICD-10: International Statistical Classification of Diseases, Tenth Revision
MGC: multidisciplinary gastrointestinal care
OR: odds ratio
PRO: patient-reported outcome
PROMIS: Patient-Reported Outcomes Measurement Information System

## Data Availability

The dataset analyzed during this study is available from the corresponding author on reasonable request.
