# Supplementary figures and images for: Virtual Multidisciplinary Gastrointestinal Care for Adults With Gastrointestinal Needs: Retrospective Cohort Study
Source: J Med Internet Res. 2026 Apr 23;28:e89061. doi: 10.2196/89061 (PMC13153750; doi:10.2196/89061)

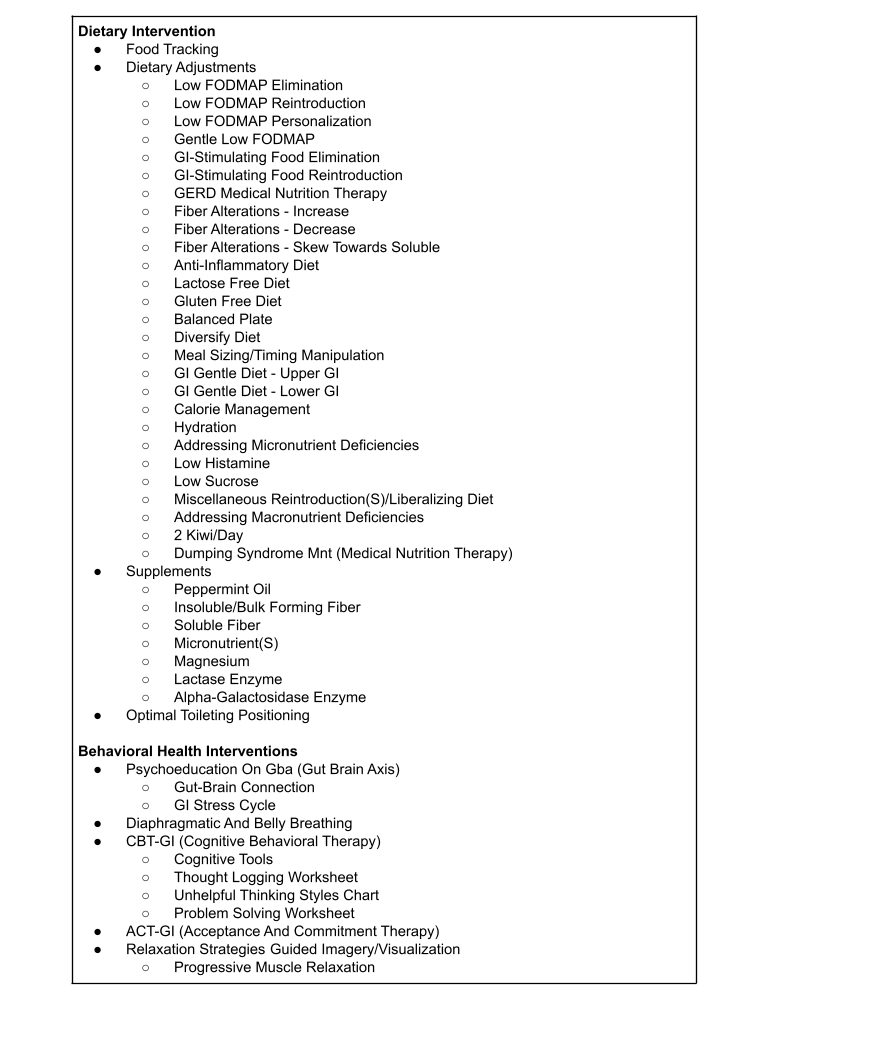

Supplement: Multimedia Appendix 1 [file jmir_v28i1e89061_app1.png]

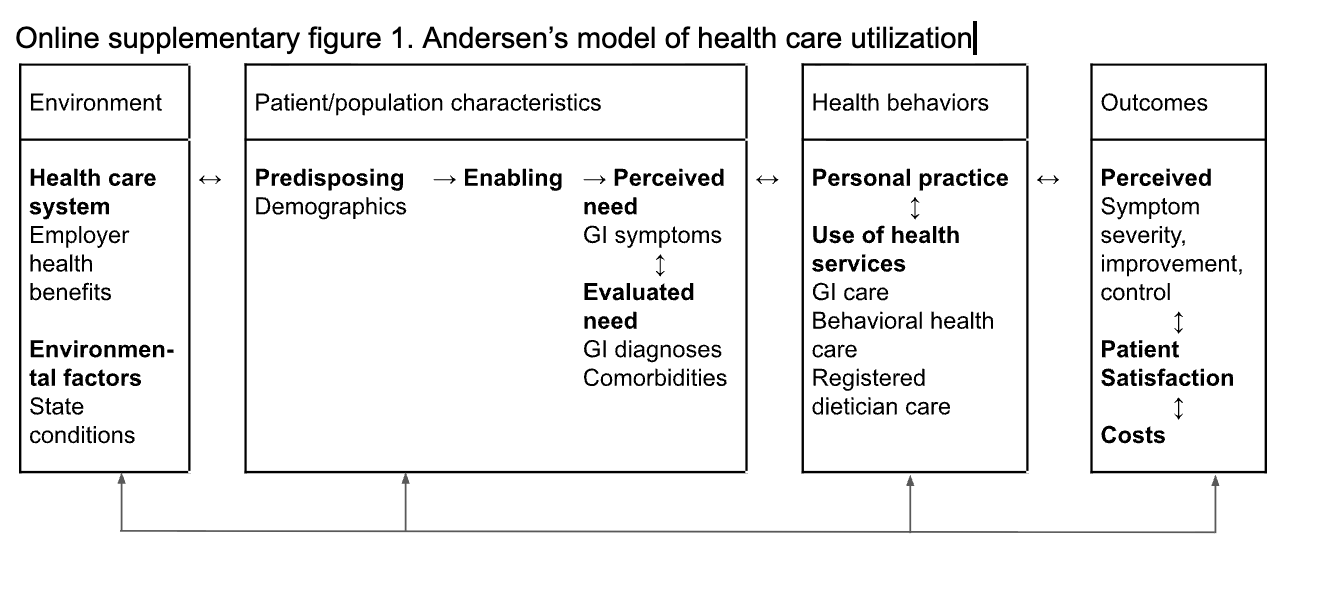

Supplement: Multimedia Appendix 2 [file jmir_v28i1e89061_app2.png]
