# Supplementary material for: Virtual Multidisciplinary Gastrointestinal Care for Adults With Gastrointestinal Needs: Retrospective Cohort Study
Source: J Med Internet Res. 2026 Apr 23;28:e89061. doi: 10.2196/89061 (PMC13153750; doi:10.2196/89061)
Supplement: Multimedia Appendix 3 [file jmir_v28i1e89061_app3.pdf]

Characteristic		Complete	Incomplete	Imputed	Total
Demographics (n, %)					
	State	11,345 (100.00%)	0 (0.00%)	0 (0.00%)	11,345 (100.00%)
	Age	11,345 (100.00%)	0 (0.00%)	0 (0.00%)	11,345 (100.00%)
	Gender	11,345 (100.00%)	0 (0.00%)	0 (0.00%)	11,345 (100.00%)
	Employer	11,345 (100.00%)	0 (0.00%)	0 (0.00%)	11,345 (100.00%)
Diagnoses (n, %)					
	DGBI (Including Ibs And Other Functional Disorders)	11,345 (100.00%)	0 (0.00%)	0 (0.00%)	11,345 (100.00%)
	GERD	11,345 (100.00%)	0 (0.00%)	0 (0.00%)	11,345 (100.00%)
	IBD	11,345 (100.00%)	0 (0.00%)	0 (0.00%)	11,345 (100.00%)
	Anal Or Rectal Fissures, Fistula, Or Abscesses	11,345 (100.00%)	0 (0.00%)	0 (0.00%)	11,345 (100.00%)
	Gastroenteritis	11,345 (100.00%)	0 (0.00%)	0 (0.00%)	11,345 (100.00%)
	Hemorrhoids	11,345 (100.00%)	0 (0.00%)	0 (0.00%)	11,345 (100.00%)
	Intestinal Malabsorption	11,345 (100.00%)	0 (0.00%)	0 (0.00%)	11,345 (100.00%)
	Diseases Of Liver	11,345 (100.00%)	0 (0.00%)	0 (0.00%)	11,345 (100.00%)
	SIBO	11,345 (100.00%)	0 (0.00%)	0 (0.00%)	11,345 (100.00%)
Symptoms (n, %)					
	Flatulence	11,345 (100.00%)	0 (0.00%)	0 (0.00%)	11,345 (100.00%)
	Bloating	11,345 (100.00%)	0 (0.00%)	0 (0.00%)	11,345 (100.00%)
	Heartburn Or Reflux Hypersensitivity	11,345 (100.00%)	0 (0.00%)	0 (0.00%)	11,345 (100.00%)
	Constipation	11,345 (100.00%)	0 (0.00%)	0 (0.00%)	11,345 (100.00%)
	Abdominal Or Pelvic Pain	11,345 (100.00%)	0 (0.00%)	0 (0.00%)	11,345 (100.00%)
	Diarrhea	11,345 (100.00%)	0 (0.00%)	0 (0.00%)	11,345 (100.00%)
	Nausea And Vomiting	11,345 (100.00%)	0 (0.00%)	0 (0.00%)	11,345 (100.00%)
	Change In Bowel Habits	11,345 (100.00%)	0 (0.00%)	0 (0.00%)	11,345 (100.00%)
	Chest Pain	11,345 (100.00%)	0 (0.00%)	0 (0.00%)	11,345 (100.00%)
	Fecal Abnormalities	11,345 (100.00%)	0 (0.00%)	0 (0.00%)	11,345 (100.00%)
	Fecal Incontinence	11,345 (100.00%)	0 (0.00%)	0 (0.00%)	11,345 (100.00%)

Characteristic		Complete	Incomplete	Imputed	Total
Patient Reported Outcome At Baseline (n, %)					
	Number Of Symptoms	11,344 (99.99%)	1 (0.01%)	1 (0.01%)	11,345 (100.00%)
	Satisfaction	10,146 (89.43%)	1,199 (10.57%)	1,199 (10.57%)	11,345 (100.00%)
	Symptom Frequency	10,942 (96.45%)	403 (3.55%)	403 (3.55%)	11,345 (100.00%)
	Symptom Severity	10,942 (96.45%)	403 (3.55%)	403 (3.55%)	11,345 (100.00%)
	Symptom Control	11,344 (99.99%)	1 (0.01%)	1 (0.01%)	11,345 (100.00%)
Engagement (n, %)					
	Number Of GI Appointments And Calls	11,345 (100.00%)	0 (0.00%)	0 (0.00%)	11,345 (100.00%)
	Number Of Behavioral Health Appointments And Calls	11,345 (100.00%)	0 (0.00%)	0 (0.00%)	11,345 (100.00%)
	Number Of Registered Dietitian Appointments And Calls	11,345 (100.00%)	0 (0.00%)	0 (0.00%)	11,345 (100.00%)
	Minutes With Behavioral Health Provider	11,345 (100.00%)	0 (0.00%)	0 (0.00%)	11,345 (100.00%)
	Minutes With GI Provider	11,345 (100.00%)	0 (0.00%)	0 (0.00%)	11,345 (100.00%)
	Minutes With Registered Dietitian	11,345 (100.00%)	0 (0.00%)	0 (0.00%)	11,345 (100.00%)
	Number Of Inbound Chat Messages	11,345 (100.00%)	0 (0.00%)	0 (0.00%)	11,345 (100.00%)
Patient Reported Outcome At Followup (n, %)					
	Symptom Severity	11,270 (99.34%)	75 (0.66%)	75 (0.66%)	11,345 (100.00%)
	Symptom Improvement	10,029 (88.40%)	1,316 (11.60%)	1,316 (11.60%)	11,345 (100.00%)
	Symptom Control	9,404 (82.89%)	1,941 (17.11%)	1,941 (17.11%)	11,345 (100.00%)
	Days To Symptom Control	8,798 (77.55%)	2,547 (22.45%)	2,547 (22.45%)	11,345 (100.00%)
	Satisfaction	5,154 (45.43%)	6,191 (54.57%)	6,191 (54.57%)	11,345 (100.00%)
